# A Novel Splice-Site Mutation in *VEGFC* Is Associated with Congenital Primary Lymphoedema of Gordon

**DOI:** 10.3390/ijms19082259

**Published:** 2018-08-01

**Authors:** Noeline Nadarajah, Dörte Schulte, Vivienne McConnell, Silvia Martin-Almedina, Christina Karapouliou, Peter S. Mortimer, Steve Jeffery, Stefan Schulte-Merker, Kristiana Gordon, Sahar Mansour, Pia Ostergaard

**Affiliations:** 1 Molecular and Clinical Sciences Institute, St George’s University of London, London SW17 0RE, UK; n.d.nadarajah@soton.ac.uk (N.N.); smartina@sgul.ac.uk (S.M.-A.); p1506900@sgul.ac.uk (C.K.); mortimer@sgul.ac.uk (P.S.M.); sggt100@sgul.ac.uk (S.J.); kristianagordon@hotmail.com (K.G.); smansour@sgul.ac.uk (S.M.); 2 Institute of Cardiovascular Organogenesis and Regeneration, Faculty of Medicine, WWU Münster, 48149 Münster, Germany; d.schulte@uni-muenster.de (D.S.); stefan.schulte-merker@ukmuenster.de (S.S.-M.); 3 CiM Cluster of Excellence (EXC1003 CiM), University of Münster, 48149 Münster, Germany; 4 Northern Ireland Regional Genetics Service, Belfast City Hospital, Belfast Health and Social Care Trust, Belfast BT9 7AB, UK; Vivienne.McConnell@belfasttrust.hscni.net; 5 South West Thames Regional Genetics Unit, St George’s University Hospitals, London SW17 0RE, UK

**Keywords:** primary lymphedema, Milroy, *VEGFC*, *VEGFR3*, *FLT4*

## Abstract

Lymphedema is characterized by chronic swelling of any body part caused by malfunctioning or obstruction in the lymphatic system. Primary lymphedema is often considered genetic in origin. *VEGFC*, which is a gene encoding the ligand for the vascular endothelial growth factor receptor 3 (VEGFR3/FLT4) and important for lymph vessel development during lymphangiogenesis, has been associated with a specific subtype of primary lymphedema. Through Sanger sequencing of a proband with bilateral congenital pedal edema resembling Milroy disease, we identified a novel mutation (NM_005429.2; c.361+5G>A) in *VEGFC*. The mutation induced skipping of exon 2 of *VEGFC* resulting in a frameshift and the introduction of a premature stop codon (p.Ala50ValfsTer18). The mutation leads to a loss of the entire VEGF-homology domain and the C-terminus. Expression of this Vegfc variant in the zebrafish floorplate showed that the splice-site variant significantly reduces the biological activity of the protein. Our findings confirm that the splice-site variant, c.361+5G>A, causes the primary lymphedema phenotype in the proband. We examine the mutations and clinical phenotypes of the previously reported cases to review the current knowledge in this area.

## 1. Introduction

Primary Lymphedema causes chronic swelling due to dysfunction within the lymphatic system. Clinical phenotyping and gene discovery has led to the development of a classification and diagnostic algorithm [1]. A congenital form of primary lymphedema represented in the algorithm is Milroy disease (also referred to as “Lymphedema, hereditary, IA”; [MIM 153100]). Milroy disease usually presents with bilateral lower limb swelling at birth. The edema is typically painless and confined to the dorsum of the foot but can extend further up the lower limb to the knees [2]. Milroy disease is an autosomal dominant condition and approximately 70% of cases are caused by mutations in *VEGFR3* (*FLT4*) with variable penetrance [3].

*VEGFR3* codes for the Vascular Endothelial Growth Factor Receptor 3, which is a tyrosine kinase-linked receptor. The Vascular Endothelial Growth Factor C (VEGFC) is the main ligand for VEGFR3 and the VEGFC/VEGFR3 signaling axis is crucial for lymphatic vascular development [4,5]. Recently, a study identified a *VEGFC* mutation in seven individuals from a three-generation family presenting with congenital lower limb lymphedema consistent with autosomal dominant inheritance [6]. This finding has been followed by two further reports of 13 individuals from three families with a similar phenotype [7,8]. These are the only reports of patients with mutations in *VEGFC* (Lymphedema, hereditary, ID, [MIM 615907] or congenital primary lymphedema of Gordon).

The pathogenic variants previously reported are a two-base pair insertion (c.571_572insTT) and a stop-gain mutation (c.C628T), which both reside in exon 4 of *VEGFC* as well as a splice-variant (c.148-3_148-2delCA) and a synonymous variant (c.552G>A) both causing exon skipping. All reported mutations are predicted to form a truncated protein, which is deemed to be dysfunctional. Functional studies in zebrafish showed that the *VEGFC* frameshift mutation (c.571_572insTT) observed in the first published family causes a loss-of-function phenotype, which suggests that the disease mechanism is haplo-insufficiency [6]).

VEGFC is synthesized as an inactive precursor molecule and activated by proteolytical processing, sequentially cleaving the N-terminal and C-terminal propeptides from the VEGF homology domain (VHD). The C-terminus of VEGFC is cleaved off by Furin [9] while the N-terminus can be removed by plasmin [10] or A disintegrin and metalloproteinase with thrombospondin motifs 3 (ADAMTS3) [11]. Proteolytic processing increases the affinity of VEGFC for VEGFR3 [12] and the extracellular matrix [13]. When fully processed, VEGFC is able to efficiently bind to VEGFR2 and VEGFR3 and activate both signaling pathways. It, therefore, has lymphangiogenic and angiogenic potential [12]. The processing of VEGFC by ADAMTS3 needs to be activated by Collagen and calcium-binding EGF domain-containing protein 1 (CCBE1) [11,14,15]. A depletion of ADAMTS3 or CCBE1 both in zebrafish and mice leads to a lack of lymphatic vessels [16,17,18].

In this paper, we report on a family with primary, congenital lymphedema where no variants were found in *VEGFR3.* As the proband presented with a congenital form of lymphedema resembling that reported by Gordon et al. [6], DNA from the proband was subjected to a *VEGFC* screen and a splice-site variant (c.361+5G>A) was identified. To investigate the likely pathogenicity of this donor splice-site mutation, further investigations were carried out. Using the same zebrafish assay described previously [6], we demonstrate that the splice-variant identified in the proband leads to reduced VEGFC activity. Our experiments demonstrate that mutant VEGFC does not interfere with wild type VEGFC in a dominant negative manner, which confirms haplo-insufficiency as the disease mechanism.

## 2. Results

### 2.1. Clinical Report

**Figure 1:**
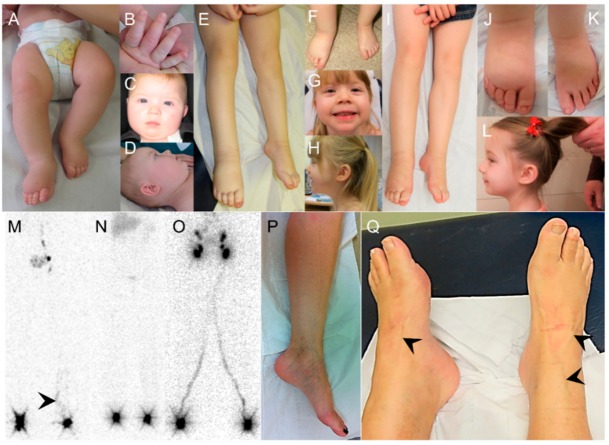
Clinical phenotype of the proband, mother and maternal grandfather and anterior view of lower limb lymphoscintigraphy. (**A**–**D**) Proband at her first visit to the genetics clinic at six months. (**A**) The proband presented as a baby with bilateral lower limb lymphedema. Swelling of the dorsum of the feet was obvious. Deep inter-phalangeal creases and small dysplastic nails were seen. (**B**) There was mild swelling in the hands. (**C**) She also had subtle dysmorphism with a round face, high forehead, short neck, hypertelorism, and depressed nasal bridge. (**D**) There was mild bilateral ear dysplasia and right pre-auricular sinus. (**E**–**H**) Proband at two years and 10 months. (**E**,**F**) The proband had persistent bilateral lower limb lymphedema of the feet, which extended up the leg to the knees and was more pronounced on the right. (**G**) Subtle dysmorphic features with a round face, hypertelorism, downslanting palpebral fissures, and depressed nasal bridge. (**H**) Slightly low, posterior rotated ears with mild ear dysplasia. (**I**–**L**) Proband at four years and eight months. (**I**) Proband presented with persistent bilateral lower limb lymphedema to the knees. (**J**,**K**) The swelling was particularly noticeable in the dorsum of the foot, which was more marked in the right foot (**J**) than the left (**K**). (**L**) Slightly low, posterior rotated ears. (**M**) Lymph scan (anterior view) of proband showing no evidence of uptake of the tracer in the right lower limb lymphatic tracts or inguinal lymph nodes after 3 h (functional aplasia). Uptake in the left lower limb lymphatic vessels remains abnormal and reduced (functional hypoplasia). The left lower leg lymphatic vessels are tortuous in the calf region (arrow head). (**N**) Lymph scan of a patient with typical Milroy disease with no evidence of main-tract filling nor rerouting (functional aplasia). (**O**) Lymph scan of healthy control. (**P**) The mother of the proband (III.1) presented with mild varicose eczema and multiple superficial veins around the ankles. There was also subtle lymphedema on the medial aspects of the ankles. (**Q**) The maternal grandfather (II.2) had bilateral mild varicose veins. There is noticeable mild lymphedema of the left ankle and varicose veins of both lower limbs (arrow heads).

The proband was born by elective caesarean section at 39 weeks with a birth weight of 3880 g (75th–91st centile) after an uneventful pregnancy. Postnatally, the proband was noted to have significant bilateral pedal edema extending to the lower tibia and was more pronounced on the right (Figure 1A). She was also noted to have bilateral mild edema of the dorsi of both hands (Figure 1B). At six months, her height was on the 25th to the 50th percentile, weight of 50th to 75th percentile and OFC (occipital frontal circumference) >9th percentile. She had subtle dysmorphism with a round face, high forehead, short neck, hypertelorism, depressed nasal bridge, mild bilateral ear dysplasia, and a right pre-auricular sinus (Figure 1C,D). There was normal development, no learning difficulties, and general health was satisfactory. She was the younger of two children born to non-consanguineous parents with a history of two previous miscarriages at five and nine weeks. Her three-year-old brother had no swelling.

At two years and 10 months, her height was in the 91st percentile, weight in the 25th to 50th percentile and OFC > 9th. There was persistent bilateral pitting edema in both feet, which extended into the lower leg on the right (Figure 1E,F). The swelling of both hands had spontaneously resolved. Her facial features still showed subtle dysmorphism with slightly low, posterior rotated ears (Figure 1G,H).

At four years and 8 months, her height was in the 25th to 50th percentile, weight in the 25th to 50th percentile and OFC > 9th. There was bilateral pitting edema in both feet and the right lower leg (Figure 1I–K) with mildly dysplastic and upslanting toenails (due to edema of the nail bed). There was no obvious facial or body asymmetry, vascular malformations, or distichiasis. She still had subtle dysmorphism with a high forehead and slightly low, posterior rotated ears (Figure 1L).

Initially a soft cardiac murmur was identified on auscultation, but subsequently an echocardiogram was reported as normal. The pelvic and renal ultrasound showed no abnormalities. Karyotype was 46,XX with no evidence of mosaicism. The array CGH (comparative genomic hybridization) was normal.

Lymphoscintigraphy performed at four years and eight months showed no uptake of tracer by the lymphatic vessels of the right lower limb and consequently no uptake within the right-sided ilio-inguinal lymph nodes, which indicated a functional aplasia (Figure 1M). The vessels in the left leg appeared tortuous in nature and were different to those seen in healthy controls and Milroy disease cases (Figure 1N,O).

The mother was a 35-year-old female with no previous medical history. However, clinically, there was evidence of varicose eczema, superficial veins, and subtle lymphedema on the medial aspects of lower legs and ankles. The patient reported that the onset was in adolescence (Figure 1P). She underwent investigation with lower limb lymphoscintigraphy, which confirmed abnormal lymphatic drainage with tortuous main lymphatic tracts.

The maternal grandfather was a 65-year-old male with a history of triple coronary artery bypass (CABG) surgery at 61 years. He had type 2 diabetes mellitus, hypothyroidism, and Perthes disease of the right hip diagnosed at five years of age, which did not require surgery. The examination revealed evidence of bilateral lower limb varicose veins, which is more obvious on the right. Subtle lower limb lymphedema was present, which is more apparent in the left lower leg (Figure 1Q) and had evidence of a surgical scar related to vein harvesting for the CABG surgery. There were bilateral venous flares around the ankles. He underwent an investigation with lower limb lymphoscintigraphy, which demonstrated abnormal lymphatic drainage. Tortuous lymphatic tracts were seen in the below-knee regions as well as an area of superficial re-routing of the tracer in the right lower leg.

The deceased maternal great-grandfather had a reported history of coronary bypass in his 70s as well as prominent veins and swelling in the lower legs.

There was no reported family history of hydroceles (fluid within the testicular sac).

### 2.2. Molecular Genetics Identifies a Mutation in VEGFC

**Figure 2:**
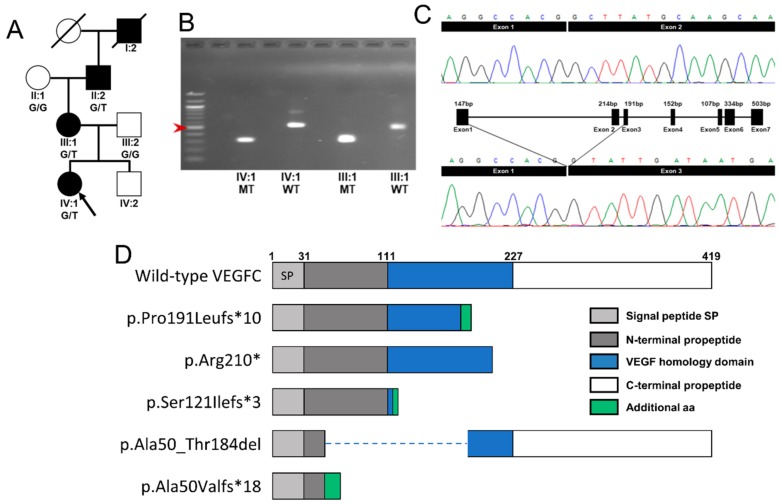
Genetic analysis of the family. (**A**) Pedigree of the family. Filled symbols denote affected individuals. Arrow indicates the proband. For individuals where DNA has been Sanger sequenced, the genotype is indicated. (**B**,**C**) After RT-PCR of RNA extracted from blood from the proband (IV:1) and her mother (III:1), cDNA amplification of a *VEGFC* fragment gave dual bands. The DNA from the bands was extracted from the gel and sequenced. (**B**) Agarose gel showing the successful separation of the wild type (WT; 489 bp) and mutant band (MT; 275 bp). Left lane: 100 bp ladder with 500 bp band is indicated with an arrow. (**C**) Electropherograms showing the *VEGFC* sequence of the products from (**B**) wild type allele (top panel) and mutant allele (bottom panel). The mutant allele highlights the skipping of exon 2 by displaying the sequence from the end of exon 1 and the beginning of exon 3. (**D**) Schematic representation of the wild type VEGFC protein domains (top) and the structures of the predicted mutant VEGFC proteins for the four previously published *VEGFC* mutations. The bottom panel shows the predicted mutant VEGFC protein described as the p.Ala50Valfs*18 identified in this family, lacking the C-terminal propeptide, the VHD, and most of the N-terminal propeptide. The additional 18 amino acids added to the sequence as a consequence of the frameshift are represented in green.

The proband had previously been screened for mutations in *VEGFR3* (exons 17–26, covering the tyrosine kinase domain) and found to be negative. DNA from the proband was then sequenced for variants in *VEGFC*. A heterozygous c.361+5G>A (g.177650682C>T, [transcript NM_005429.2]) variant was identified in the intronic region downstream of exon 2 of the proband (IV:1). The same variant was identified in the mother (III:1) and the maternal grandfather of the proband (II:2) (Figure 2A). Mutation Taster suggested that this variant could be pathogenic with an effect on splicing. Human Splicing Finder reported that the variant could cause an alteration of the donor splice site in intron 2.

To analyze the consequence of the c.361+5G>A mutation, the cDNA of the proband and her mother was amplified using primers specific for *VEGFC*. Two bands were identified in the gel representing the product of wild type mRNA (489 bp) and the product of mutant mRNA (275 bp). The 214 bp loss in the mutant band suggested skipping exon 2 in the mutant form. Sequencing of the wild type and mutant bands extracted from the agarose gel confirmed that exon 2 is spliced out during the transcription of the mutant allele of the proband and her mother (Figure 2B,C). The verified mRNA change (r.148_361del) is likely to cause the production of a premature stop codon, which is suggested to result in a truncated protein described as p.Ala50ValfsTer18. Therefore, the resultant protein is predicted to consist of only part of the N-terminal propeptide, with a complete loss of the VEGF homology domain and the C-terminal propeptide (Figure 2D). The variant is available in the Leiden Open Variation Database (http://databases.lovd.nl/shared/genes/VEGFC) with the variant accession ID: 195638.

### 2.3. Vegfc Variant Fails to Promote Vessel Sprouting in Zebrafish

**Figure 3:**
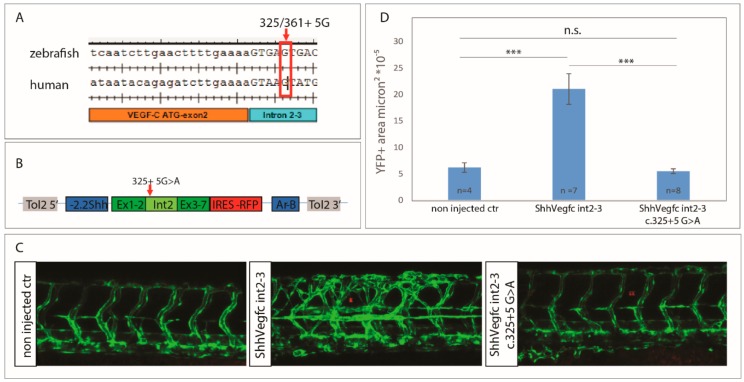
Splice site mutation c.325+5G>A in *vegfc* abolishes the effect of ectopic expression on excessive vessel sprouting. (**A**) Sequence of the human and zebrafish *VEGFC/vegfc* orthologs. Alignment of zebrafish and human exon 2 and intron 2–3 highlights the mutated site c.361+5G, which is equivalent to zebrafish c.325+5G. (**B**) Diagram of constructs used for forced expression of either vegfc-intron2–3 wt or vegfc-intron2–3 c.325+5G>A together with tagRFP in the floorplate, which consists of a 5′ and 3′ Tol2 element, the -2.2Shh promoter region, exon 1–2 of the cDNA of *vegfc* (Ex1–2), vegfc-intron 2–3 (Int 2) of *vegfc* (wt or c.325+5G>A), exon 3–7 of *vegfc* (Ex3–7), an IRES tagRFP cassette, and the activating region Ar-B driving expression in the floorplate. (**C**) Analysis of vegfc-intron2–3 wt and vegfc-intron2–3 c.325+5G>A overexpression in the floorplate using the transgenic line *Tg(flt4^BAC^:mCitrine)^hu7135^* at 56 hpf. Overexpression of vegfc-intron 2–3 wt led to lymphovenous hypersprouting while forced expression of vegfc-intron2–3 c.325+5G>A had no effect on vessel growth. **(D)** Quantification of the YFP positive area of vessels surrounding the site of tagRFP expression showed an increased vessel density in zebrafish overexpressing vegfc-intron 2–3 wt but not in zebrafish overexpressing the vegfc-intron 2–3 c.325+5G>A variant. Values are presented as means ± standard error of mean values (SEM). *** *p* < 0.001, n.s. not significant.

To analyze the effect of the c.361+5G>A mutation in vivo, we overexpressed Vegfc in the floorplate of zebrafish. In zebrafish, Vegfc/Vegfr3 signaling is crucial for lymphovenous sprouting and the development of the lymphatic system [19,20,21]. The donor splice site of intron 2–3 between zebrafish and human is conserved (Figure 3A). Zebrafish *vegfc c.325+5G* is equivalent to human *VEGFC c.361+5G*. In the in vivo assay, we overexpressed zebrafish *vegfc* cDNA including intron 2–3 (vegfc-intron2–3) together with tagRFP to monitor expression of the transgene (Figure 3B). In this assay, we would expect the wild type (wt) to stimulate excessive vessel sprouting while a mutation which has an effect on protein function, reduces or inhibits vessel sprouting [6]. In our experiment, the expression of vegfc-intron2–3 wt led to excessive vessel sprouting while expression of the mutant vegfc-intron2–3 c.325+5G>A had no effect on the vasculature (Figure 3C,D). Therefore, the mutation significantly reduces, or possibly removes, the biological activity of the protein.

### 2.4. Vegfc Splice Variant Leads to Production of a Truncated Protein

**Figure 4:**
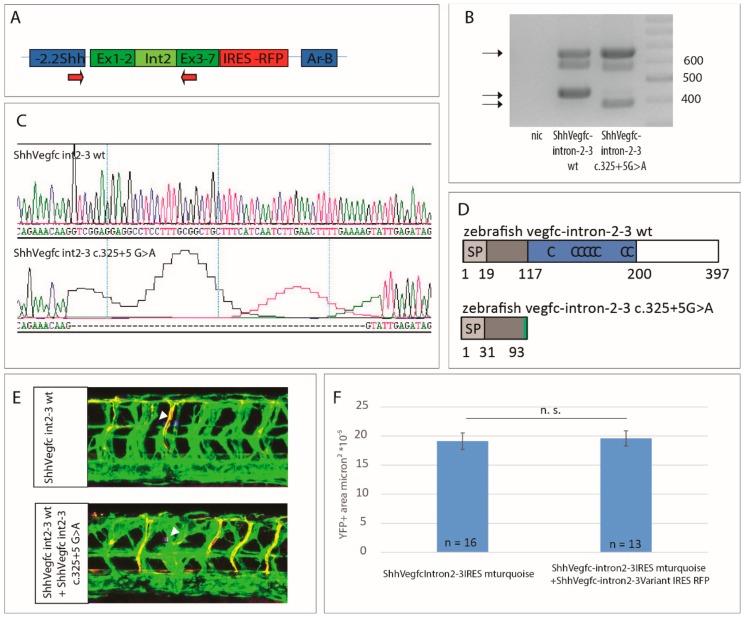
The *vegfc c.325+5G>A* splice-site variant results in a truncated protein, which does not have dominant negative activity. (**A**) Diagram of the construct used for overexpression of vegfc-intron 2–3 depicting primers used for RT-PCR (arrows). (**B**) RT-PCR of zebrafish embryos expressing ShhVegfc-intron 2–3 wt or ShhVegfc-intron 2–3 c.325+5G>A. Embryos expressing the wt form of ShhVegfc-intron 2–3 and the ShhVegfc-intron 2–3 c.325+5G>A variant express a band of 660 bp corresponding to non-spliced RNA or integrated plasmid DNA as well as a second smaller band corresponding to wt ShhVegfc (440 bp) or mutant ShhVegfc (387 bp). Right lane: 100 bp ladder. Non-injected control, nic. (**C**) Sequencing of cDNA of embryos expressing ShhVegfc-intron 2–3 and the ShhVegfc-intron 2–3 c.325+5G>A variant showing a 53 bp deletion in the ShhVegfc-intron 2–3 c.325+5G>A variant (lower panel). (**D**) Schematic representation of predicted wild type (top) and mutant (bottom) proteins. Mutant protein consists of the first 91 amino acids of Vegfc containing only a part of the N-terminus of Vegfc but not the VHD or the C-terminus (see Figure 2 for legend). (**E**) Analysis of co-overexpression of ShhVegfc-intron 2–3 wt and ShhVegfc-intron2–3 c.325+5G>A in the floorplate using the transgenic line *Tg(flt4^BAC^:mCitrine)^hu7135^* and *Tg(flt1^enh^:tdTomato)*, which marks venous and lymphatic cells in green and arterial vessels in red at 48 hpf. Expression of ShhVegfc-intron 2–3 wt and ShhVegfc-intron 2–3 c.325+5G>A within the same cell are monitored by the simultaneous expression of mturquoise and tagRFP, respectively. Forced expression of ShhVegfc-intron 2–3 wt in the floorplate led to excessive vessel sprouting comparable with co-overexpression of ShhVegfc-intron 2–3 wt and ShhVegfc-intron 2–3 c.325+5G>A in the floorplate. Arrow heads mark expression of tagRFP and mturquoise in the floorplate. (**F**) Quantification of lymphovenous sprouting by measuring the YFP positive area surrounding the site of mturquoise/tagRFP expression. Values are presented as means ± standard error of mean values (SEM). n.s. not significant.

To analyze the effect of the mutation on the Vegfc protein, we analyzed the splicing of the cDNA of *vegfc* including intron 2–3 in comparison with the *vegfc* variant c.325+5G>A. To this end, we used primers binding in the 5′UTR of the ShhVegfc-intron2–3 IRES tagRFP transgene and in exon 3 of *vegfc* (Figure 4A). Zebrafish embryos expressing wt vegfc-intron 2–3 or vegfc-intron 2–3 c.325+G>A both express a band of 660 bp corresponding to non-spliced cDNA or integrated plasmid DNA (Figure 4B). The spliced cDNA of ShhVegfc-intron 2–3 is visible as a band of 440 bp in zebrafish embryos expressing ShhVegfc-intron 2–3 wt (Figure 4B). Zebrafish embryos expressing the vegfc-intron 2–3 c.325+5G>A variant show a band of slightly smaller size (387 bp) corresponding to a *vegfc* cDNA lacking 53 bp of exon 2 (Figure 4B), which is also evident from the sequencing of this cDNA (Figure 4C). This deletion leads to a frameshift mutation producing an additional amino acid (Y) followed by a stop codon in the Vegfc protein (Figure 4D). The resulting protein consists of only the first 91 amino acids of Vegfc with only part of the N-terminus of Vegfc but missing the VEGF homology domain and the C-terminus.

### 2.5. The Vegfc-intron 2–3 c.325+5G>A Variant Does Not Have Dominant Negative Activity

Next, we tested whether the mutant Vegfc variant has dominant negative activities. As such, we co-overexpressed vegfc-intron 2–3 c.325+5G>A and vegfc-intron 2–3 wt in the zebrafish floorplate. Expression of both transgenes in the same cell was assured using IRES tagRFP and IRES turquoise cassettes, respectively. An equal amount of hypersprouting could be observed between embryos expressing vegfc-intron 2–3 wt only and embryos co-expressing vegfc-intron 2–3 wt and the vegfc-intron 2–3 c.325+5G>A variant (Figure 4E,F). Therefore, we conclude that the vegfc-intron 2–3 c.325+5G>A variant does not result in a protein with dominant negative properties but most likely leads to a haplo-insufficient phenotype.

## 3. Discussion

Congenital primary lymphedema associated with VEGFC is a rare primary lymphedema with only three, previously reported studies [6,7,8]. In this study, we provide independent evidence that *VEGFC* mutations are causative of primary lymphedema of the type originally described by Gordon et al. [6]. In this non-consanguineous family with autosomal dominant inheritance (consistent with the inheritance pattern seen in similar cases), we have identified an unreported pathogenic *VEGFC* variant.

Congenital primary lymphedema of Gordon typically presents, like Milroy disease, with congenital, pedal lymphedema with prominent, wide caliber veins over the shins and on the dorsa of the feet. The patients have upslanting, dysplastic toenails and deep, interphalangeal creases of the toes. In this manuscript, we report on a proband who presented with a congenital form of lymphedema resembling that of Gordon et al. [6]. Albeit mild, the proband also had lymphedema of the dorsi of both hands post-natally, which is a little unusual. However, it did not require significant intervention and spontaneously resolved with time.

The extent of the lymphedema varies within the family with the mother of the proband being very mildly affected and the grandfather moderately affected. Of the 23 individuals (11 male and 12 female) from 5 families (including the family described here) reported to date, we see marked inter-familial and intra-familial variation (Table 1). For example, 17 of the 23 individuals (74%) have some forms of swelling in which most had bilateral below-knee lymphedema (*n* = 12) but some cases had isolated ankle and foot lymphedema (*n* = 5). There were also reports of reduced penetrance since six individuals carrying a *VEGFC* mutation had no clinical signs of primary lymphedema (*n* = 4) or reported occasional swelling (*n* = 2) of feet and ankles.

The lymphedema usually presents at birth but age of onset can vary. The majority of individuals (70%, nine of the 13 where the age of onset has been recorded) presented with congenital (<1 year) primary lymphedema. Of other clinical signs, 27% (3/11) of the males presented with hydrocele. The second most common finding after lymphedema in males with Milroy disease was hydrocele, which was reported in 37% of males [2]. They also reported prominent veins in 23% of individuals with Milroy disease. Not all the reports on *VEGFC* cases confirm the absence or presence of prominent veins but, in the nine individuals where a note has been made, five of those (55%) had such veins. There are too few reports on *VEGFC* cases yet to confirm whether prominent veins are a more common feature in congenital primary lymphedema of Gordon compared to Milroy disease.

The lymphoscintigraphy imaging of the proband and the grandfather showed unusual lymphatic tracts, which corresponded to the other lymphoscintigraphies reported for this condition (Table 1). Although the mother (III:1) and grandfather (II:2) presented with very mild clinical signs of lymphedema, reduced uptake of a tracer in the groin area was reported for both, which indicated that the clinical presentation does not always represent the physiological situation. This would suggest that mutation carriers who appear to have no clinical signs of lower limb swelling could still have a compromised lymphatic system. Of the 23 individuals reported in Table 1, nine have undergone lymphoscintigraphy imaging and, of those, one was reported as a *VEGFC* mutation carrier with no sign of lower limb lymphedema (Family 1, III:2) but had tortuous tracts on the lymph scan.

In summary, the cases with *VEGFC* mutations where lymphoscintigraphy has been performed all show some uptake in the groin at a reduced rate. The tracer is following tortuous tracts with rerouting around feet, ankles, and lower legs, which indicates that the tracer is not following the normal tracts to the groin area seen in healthy controls. This contrasts with the lymphoscintigrams seen in Milroy disease, which show no uptake within the main lymphatic tracts and groin area after 2 h. This suggests initial lymphatic vessel dysfunction. Therefore, lymphoscintigraphy could prove a useful tool to differentiate between the two primary lymphedema phenotypes.

We describe a novel splice-site mutation (c.361+5A>G) in *VEGFC*, which co-segregates with the disease phenotype in the family. We have shown that exon 2 was spliced out of the mRNA. This is predicted to cause a frameshift and premature truncation of the protein, which produces a polypeptide lacking the VEGF homology domain and the C-terminal domain. The results from our zebrafish study corroborate this finding since the effect of the mutation on zebrafish Vegfc is also predicted to encode a severely truncated protein.

The truncated VEGFC protein in this family has a proposed protein size of 69 amino acids, which is the smallest reported in patients with a similar phenotype. The previously reported variants are an indel, a stop-gain mutation and two variants causing defective splicing (Table 1). All four variants are predicted to result in a truncated protein (Figure 2D). According to ExAC, the number of loss of function variants compared to missense variants in *VEGFC* is very low and the tolerance to loss of function mutations is predicted to be very low (pLI = 0.12). This means that the resultant proteins in the five families are deemed non-functional.

Studies have shown that the loss of the C-terminal domain is detrimental to the cleavage of the peptide [13]. In addition, phosphorylation of the tyrosine kinase domain in VEGFR3 is dependent on the presence of the VEGFC VHD [12]. Therefore, the effect of the observed variant on splicing could be a direct cause of disease in the family presented in this study since the protein produced lacks the VHD and the C-terminal propeptide. The other four truncated proteins (Figure 2D) also lack a complete VHD, which is most likely going to affect the phosphorylation of VEGFR3. In addition, most of the mutant proteins also lack the C-terminal propeptide. Therefore, proper cleavage of VEGFC is also disrupted. This occurs if the protein is being secreted to the extracellular matrix where the cleavage takes place. The work of Gordon et al. showed that, although the truncated mutant protein c.571_572insTT is stable, it is not secreted efficiently [6]. Therefore, the VEGFR3 activity is affected and downstreamVEGFR3 signaling pathways are disrupted, which is expected to lead to a lymphatic phenotype if the effect is comparable to that of Milroy disease associated *VEGFR3* mutations.

We showed previously that the reported mutation c.571_572insTT can disrupt the biological activity of the Vegfc protein [6]. Using a similar Vegfc induced sprouting assay in zebrafish, we have demonstrated that the c.361+5A>G splice-site variant reported in this manuscript also gives rise to a protein with very reduced or no biological activity. The lack of protein activity was represented by a lack of sprouting of lymphatic vessels in our assay. Inactivation of Vegfc in various animal models (mice, zebrafish, xenopus tadpoles) shows that lymphatic endothelial cells differentiate in the cardinal vein, but the Prospero homeobox protein 1 (PROX1) positive cells fail to bud off and migrate [5,21,22,23].

We wanted to determine whether the disease mechanism is dominant negative since it has been shown for VEGFR3 [4,24]. In this assay with the c.361+5A>G mutation, we could not detect dominant negative activity of the N-terminal propeptide of Vegfc. This corroborates the findings for the c.571_572insTT [6]. Mice null for *Vegfc* die [5,25], but heterozygous *Vegfc* mice and *Chy3* mice (hemizygous for *Vegfc*) survive to adulthood but with edematous paws and dermal hypoplasia [5,26], which confirms haplo-insufficiency as the disease mechanism. Therefore, we conclude that the human patients are likely to present a haplo-insufficient phenotype.

As mentioned, haplo-insufficient *Vegfc* mice resemble *Chy3* mice and both strains have edema of variable severity. Likewise, *Chy* mice, which have a heterozygous germline mutation (a p.Ile1053Phe substitution) in the Vegfr3 tyrosine kinase domain, show a similar phenotype [24]. Therefore, the three mouse models are very similar to the human phenotypes of the *VEGFR3*-associated Milroy disease and the *VEGFC*-associated congenital primary lymphedema of Gordon. Nevertheless, to date, only five different mutations in the *VEGFC* gene in patients with congenital primary lymphedema of Gordon have been identified while the number of identified mutations in the *VEGFR3* gene in Milroy disease patients is much higher (58 different variants in 95 families as summarized by Gordon et al. [27]). Of these cases, patients with *VEGFC* mutations demonstrate impaired lymphatic drainage on lymphoscintigraphy imaging. However, lymphatic function is greater than that seen in patients with *VEGFR3* mutations who demonstrate profound abnormalities with a functional aplasia of the lymphatic system. One possible explanation of a mild etiology of congenital primary lymphedema of Gordon is the possible compensation for the loss of VEGFC by other factors such as VEGFD since the mature form of human VEGFD binds both VEGFR2 and VEGFR3 [28,29]. This is entirely possible since it has been demonstrated that Vegfd can compensate for the loss of Vegfc in mouse [5,30] and zebrafish [31]. Overexpression of VEGFD in *Vegfc* heterozygous mice can rescue the lymphatic phenotype [30].

A combined deletion of both *Vegfc* and *Vegfd* in mice does not mirror the phenotype observed in mice deleted for *Vegfr3*, which suggests that other factors can activate VEGFR3 [30]. Ligand-independent VEGFR3 activation has been proposed in several studies [32,33,34]. Therefore, other unknown ligands or ligand-independent signaling via VEGFR3 or interaction of VEGFR3 with other factors may explain why mutations in *VEGFC* are responsible for a milder phenotype. Further studies are required to fully understand the biochemical interactions between the proteins in the VEGFR3/VEGFC signaling axis and their role in causing primary lymphedema.

In summary, most patients with Milroy disease have pathogenic variants in the *VEGFR3* gene, but mutations in the ligand known as *VEGFC* can also give rise to a very similar disorder. Although rare, this should be investigated in any cases of Milroy disease screening negative for *VEGFR3* mutations especially if the lymphoscintigraphy differs from that of Milroy disease. From a clinical perspective, the key message is that congenital primary lymphedema of Gordon presents in the same manner as Milroy disease. However, the degree of lymphatic impairment is milder. This is evidenced clinically with slightly less severe lower limb lymphedema and by the lymphoscintigraphies demonstrating significantly more uptake of tracers within lymphatic vessels compared to those in Milroy disease. With this report, it has also been shown that splice-site variants in *VEGFC* should be considered as a causative agent of disease, which corroborates the findings of Fastre et al. [8]. While only three previous studies have been published, it is clear that there is intra-familial variation in the age of onset and severity of lymphedema. When a child presents with congenital pedal lymphedema, very careful evaluation of the parents is recommended. Moreover, venous disease is a consistent feature in the affected individuals.

Future work should be conducted to further elucidate the interaction of VEGFC and VEGFR3 as well as to try and identify the reason for inter-familial and intra-familial variation in both Milroy disease and congenital primary lymphedema of Gordon.

## 4. Material and Methods

### 4.1. Patient Recruitment

A proband with congenital, bilateral pedal lymphedema with no identifiable mutations in *VEGFR3* was selected for study. Ethical approval was given by the South West London Research Ethics Committee (REC Ref: 05/Q0803/257, 08/09/2006). Written informed consent was obtained from all participants. The proband and her family members underwent a detailed clinical examination.

### 4.2. Lymphoscintigraphy

Lymphoscintigraphy is the imaging of the lymphatic system by injecting radioactive isotope (technetium-99m) into the web spaces between the toes and/or fingers and imaging the uptake into the inguinal lymph nodes for foot injections after 30 min and 2–3 h.

### 4.3. PCR and Direct DNA Sequencing of the Human VEGFC Gene

DNA from the proband was analyzed for sequence variants in the *VEGFC* gene, which was previously described [3]. PCR products were sequenced using BigDye Terminator v3.1 chemistry (Life Technologies, Carlsbad, CA, USA) and an ABI3130xla Genetic Analyzer (Life Technologies). Sequencing traces were visually inspected in FinchTV v1.4 (Geospiza) and aligned with a reference sequence using CLC Sequence Viewer (CLC bio-Qiagen, Qiagen, Hilden, Germany). The identified variant was checked in dbSNP (www.ncbi.nlm.nih.gov/SNP/), 1000 Genomes Project (www.1000genomes.org), and gnomAD (www.gnomad.broadinstitute.org) for novelty. The variant was also analyzed in Mutation Taster [35] and Human Splicing Finder [36] for genetic effect. For co-segregation analysis, PCR and sequencing were carried out in the parents and maternal grandparents of the proband.

### 4.4. RNA/cDNA Analysis of the Splice Variant

Blood was collected from the proband and the mother of the proband using PAXgene blood RNA Tubes (PreAnalytix, Hombrechtikon, Switzerland). Total RNA was extracted using the PAXgene blood RNA Kit (PreAnalytix) and DNAse treatment performed to eliminate genomic DNA. cDNA was synthesized from the RNA with a SuperScript II Reverse Transcriptase using about 400 ng RNA and 50 ng random primers (Invitrogen, Carlsbad, CA, USA). Touchdown Hot Start PCR was carried out on the cDNA using primers which span exon 1 to exon 4 of *VEGFC* (NM_005429.2): 1aF 5′-GTCCTTCCACCATGCACTTG-3′ and 4aR 5′-GCTGGCAGGGAACGTCTAAT-3′. After the first PCR, a nested PCR was conducted using primers that span from exon 1 to exon 3: 13F 5′-CGTGTTCTCTGCTCGCCG-3′ and 13R 5′-CACTGCAGCCCCTCACTA-3′. The PCR products were run on a 1.5% Agarose gel (Geneflow A4-0700) and extracted from the gel with the Monarch DNA Gel Extraction Kit (New England BioLabs Inc., Ipswich, MA, USA). Sequencing was carried out as described above. The protein prediction tool Expasy (www.expasy.org) was used to confirm the effect of the splice-site variant on the protein sequence.

### 4.5. Cloning of the Zebrafish Vegc Expression Plasmids

Zebrafish vegfc-intron 2–3 was amplified from genomic DNA of TL zebrafish using primers zvegfc+int2–3for (5′GCAGTTGCGTTCAGCGGGTAGTGT3′) and zvegfc+int2–3rev (5′GCTGATGTATGAAGTGCTGATGTT3′) and cloned into the zebrafish *vegfc* cDNA sequence using *StuI* and *SphI* restriction enzymes. This leads to a construct comprising exons 1–2 followed by intron 2–3 and by exons 3–7 of *vegfc* (vegfc-intron 2–3 wt). The mutant vegfc-intron 2–3 c.325+5G>A variant was generated by amplifying the coding sequence of vegfc-intron 2–3 in a pCS2 vector, according to the QuikChange Site-Directed Mutagenesis protocol (Stratagene) using the primer pair 5′CTTTTGAAAAGTGAaTGACTATTAATTTAGAACCGCC3′ and 5′GGCGGTTCTAAATTAATAGTCAtTCACTTTTCAAAAG3′. An IRES site followed by tagRFP or mturquoise was introduced downstream of the vegfc-intron 2–3 and vegfc-intron 2–3 c.325+5G>A in pCS2. For expression of vegfc-intron 2–3 in the zebrafish floor plate, the vegfc-intron 2–3 cDNAs were each cloned into a plasmid containing the sonic hedgehog promoter and a floor plate specific enhancer (Ar-B) [37] flanked by MiniTol2 sites [38].

### 4.6. Zebrafish Sprouting Assay

All zebrafish strains were maintained at the University of Münster using standard husbandry conditions and following the guidelines of the animal ethics committee at the University of Münster, Germany. The transgenic reporter line *Tg(flt4^BAC^:mCitrine)^hu7135^* marking blood and lymphatic endothelial cells and the transgenic reporter line *Tg(flt1^enh^:tdTomato)* were used in this study [39,40].

Ectopic overexpression of Vegfc in the zebrafish floorplate was driven by a sonic hedgehog promoter and a floorplate specific activator region [37]. Expression of Vegfc was monitored by simultaneous expression of tagRFP or mturquoise [6]. Plasmids encoding the vegfc-intron 2–3 cDNA or vegfc-intron 2–3 c.325+5G>A. The floorplate specific promoter and enhancer regions flanked by MiniTol2sites were co-injected at 25 ng/µL together with tol2 transposase mRNA (25 ng/µL) into zebrafish eggs at the 1–2 cell stage. Embryos were selected at 2 dpf for comparable expression of tagRFP or mturquoise and imaged at 48 or 56 hpf on a Leica SP8 confocal microscope. For quantification of vessel sprouting, black and white threshold images of an area of 150 × 250 microns around the tagRFP or mturquoise positive area were produced in order to quantify the sum of the YFP+ (Yellow Fluorescent Protein positive) area of all single z-planes using ImageJ (NIH, Bethesda, MD, USA). Data sets were tested for normality (Shapiro-Wilk) and equal variance. *p*-values were determined by a Student’s *t*-test. Values are presented as means ± standard error of mean values (SEM). *** *p* < 0.001.

### 4.7. Generation of cDNA from Zebrafish Embryos

RNA of zebrafish embryos was isolated at 56 hpf using the RNeasy Plus kit from Qiagen following the manufacturer’s instructions. Reverse transcription was performed using random hexamers and M-MULV (Promega, Madison, WI, USA). cDNA spanning the 5′ UTR of the Shh promoter plasmid to exon 3 of *Vegfc* was amplified using the primers 5′TTCCCCACATCTAAACAAACT3′ and 5′CCGCGCCTCGACAACGAGAAACCCTGCTA3′ and sequenced with the same primers using Sanger sequencing.

## Figures and Tables

**Table 1:** Summary of findings of *VEGFC* mutation positive patients published to date. Family 5 is the current case. BLL, bilateral lower limb lymphedema extending to below knee. FA, lymphedema confined to feet and ankles. L, left. LL, lower limb lymphedema extending below the knee. N, not present. occ, occasional. R, right. VV, varicose veins. Y, yes. - not recorded or not applicable. Family 1 [6]. Family 2 [7]. Family 3 and Family 4 are published as LE-627 and LE-445, respectively, in Fastre et al. [8]. Family 5: this case report.

	Patient	Gender	Age of Onset	Age at Last Clinical Examination	Lymphedema at Last Clinical Examination	Hydrocele	Prominent Veins around Ankles and Feet	Lymphoscintigraphy Result			*VEGFC* Mutation	Comment
								Reduced Uptake	Tortuous Tracts	Rerouting		
Family 1	I:2	F	<5 years	-	BLL	-	-	RL	RL	N	c.571_572insTT p.Pro191Leufs*10	Venous flares and telangiectasia
	II:1	M	12 years	18 years	N	Y	Y	N	RL	N		
	II:3	F	at birth	28 years	BLL	-	Y	L	R	L		Edema improved spontaneously in childhood but deteriorated in adolescence
	II:4	M	at birth	32 years	BLL L>R	N	N	L>R	N	RL		
	III:1	F	6 months	6 months	FA	-	N	-	-	-		
	III:2	M	at birth	3 years	FA	N	Y	-	-	-		Swelling improved at 3y
	III:3	F	-	5 years	N	-	Y	-	-	-		
Family 2	IV:4	F	at birth	20 months	LL L	-	-	-	-	-	c.628C>T p.Arg210X	BLL L > R at birth
	III:3	M	at birth	38 years	LL R	Y	-	R	-	Y		
	II:2	F	>30 years	-	LL L	-	-	-	-	-		Recurrent miscarriages
Family 3	III:1	M	-	22 years	BLL	Y	-	RL	N	L>R	c.148–3_148–2delCA r.148_552del p.Ala50_Thr184del	Diagnosed at 7 years
	II:3	M	-	-	N	N	-	-	-	-		
Family 4	V:1	M	at birth	-	BLL	N	-	-	-	-	c.552G>A r.362_552del p.Ser121Ilefs*3	
	V:2	M	at birth	-	BLL	N	-	-	-	-		
	V:3	F	-	-	N	-	-	-	-	-		
	IV:2	F	-	-	occ FA	-	-	-	-	-		
	IV:3	F	-	-	FA	-	-	-	-	-		
	III:2	F	-	-	occ FA	-	-	-	-	-		
	III:5	M	-	-	FA	N	-	-	-	-		
	IV:8	M	-	-	FA	N	-	-	-	-		
Family 5	IV:1	F	at birth	4 years 8 months	LL R, FA L	-	N	R	L	L	c.361+5G>A r.148_361del p.Ala50Valfs*18	Mild bilateral edema of hands at birth, which spontaneously resolved
	III:1	F	>13 years	35 years	mild BLL	-	Y	RL	RL	N		
	II:2	M	-	65 years	mild BLL R>L	N	VV R>L	RL	RL	R		
